# Risk factors and clinical significance of lower perigastric lymph node metastases in Siewert type II and III esophagogastric junction adenocarcinoma: a retrospective cohort study

**DOI:** 10.1007/s00464-024-10875-y

**Published:** 2024-05-31

**Authors:** Xinyu Qi, Maoxing Liu, Kai Xu, Fei Tan, Pin Gao, Zhendan Yao, Nan Zhang, Hong Yang, Chenghai Zhang, Jiadi Xing, Ming Cui, Xiangqian Su

**Affiliations:** 1 Key Laboratory of Carcinogenesis and Translational Research (Ministry of Education), Department of Gastrointestinal Surgery IV, Peking University Cancer Hospital & Institute, Beijing, 100142 People’s Republic of China; 2 State Key Laboratory of Holistic Integrative Management of Gastrointestinal Cancers, Beijing Key Laboratory of Carcinogenesis and Translational Research, Department of Gastrointestinal Surgery IV, Peking University Cancer Hospital & Institute, Beijing, China

**Keywords:** Lower perigastric lymph node, Risk factors, Efficacy index, Lymph node metastases

## Abstract

**Background:**

No consensus has been concluded with regarding to the scope of lymph node (LN) dissection for Siewert type II and III adenocarcinoma of the esophagogastric junction (AEG). This study aimed to explore risk factors for lower perigastric LN (LPLN) metastases (including no. 4d, 5, 6, and 12a LN stations) and analyze the indications for LPLN dissection.

**Methods:**

In total, 302 consecutive patients with Siewert type II and III AEG who underwent total gastrectomy (TG) were enrolled. The logistic regression model was used to perform uni- and multivariate analyses of risk factors for LPLN metastases. Kaplan–Meier curves were used for survival analysis, and log-rank tests were used for group comparisons. Basing on the guidelines of Japanese Gastric Cancer Association, the LN metastases (LNM) as well as the efficiency index (EI) of each LN station was further evaluated.

**Results:**

The independent risk factors for LPLN metastases in patients with Siewert type II and III AEG were distance from the esophagogastric junction (EGJ) to the distal end of the tumor (> 4.0 cm), preoperative carcinoembryonic antigen (CEA) ( +), pT4 stage, and HER-2 ( +). LPLN metastases was an independent risk factor for overall survival following TG. The LNM and EI of LPLN were 8.6% and 2.31%, respectively. The LNM of LPLN > 10% under the stratification of the distance from the EGJ to the distal end of the tumor (> 4.0 cm), pT4, preoperative CEA ( +), and HER-2 ( +) exhibited EI values of 3.55%, 2.09%, 2.51%, and 3.64%, respectively.

**Conclusions:**

LPLN metastases was a malignant factor for the prognosis of patients with Siewert type II and III AEG. For patients with preoperative CEA ( +), pT4 stage, HER-2 ( +), and the distance from the EGJ to the distal end of the tumor (> 4.0 cm), TG with LPLN dissection is prioritized for clinical recommendation.

**Supplementary Information:**

The online version contains supplementary material available at 10.1007/s00464-024-10875-y.

In recent years, there has been a notable increase in the global incidence and mortality of adenocarcinoma of the esophagogastric junction (AEG), particularly in Asia [1, 2]. Gastroesophageal reflux disease and Helicobacter pylori eradication have been identified as the primary causes of AEG [3]. The Siewert classification, based on tumor epicenters, is widely employed for categorizing AEG: type I tumors are located 5 to 1 cm above the esophagogastric junction (EGJ), type II tumors extend from 1 cm above to 2 cm below the EGJ, and type III tumors are situated 2 to 5 cm below the EGJ [4]. Surgical intervention remains the cornerstone of AEG treatment, with subtotal esophagectomy combined with proximal gastrectomy (PG) being the standard approach for type I AEG [5]. However, optimal surgical strategies for type II and III AEG remain contentious. For type II AEG, where tumors are centered at the EGJ, the extent of lymph node metastases (LNM) correlates with the extent of esophageal and gastric invasion [6]. Consequently, thoracic surgeons often favor transthoracic esophagectomy (TTE), while gastrointestinal surgeons lean towards transhiatal total gastrectomy (THTG). With regards to type III AEG, there is a trend among gastrointestinal surgeons to transition from total gastrectomy (TG) to PG, influenced by the concept of pylorus-preserving gastrectomy. Comparative studies have shown similar 5-year overall survival rates between TTE and THTG, but the oncological outcomes of TG and PG require further investigation. The key distinction among these surgical approaches lies in the decision to dissect the lower perigastric lymph node (LPLN) for Siewert type II and III AEG. Consequently, the choice between TG and PG for Siewert type II and III AEG hinges on whether LPLN dissection is deemed necessary.

The advantages of standard D2 lymph node (LN) dissection for gastric cancer treatment have been widely acknowledged and advocated. According to gastric cancer guidelines, LPLN encompasses LNs in groups no. 4d, 5, 6, and 12a, which must be dissected in TG for Siewert type II and III AEG [7]. However, multicenter retrospective and prospective studies conducted by the Japanese Gastric Cancer Association (JGCA) and Japan Esophageal Society have indicated relatively low rates of LPLN metastases in AEG and limited benefits associated with dissection [2, 6]. Some studies have even suggested that when LPLN metastases occurs, the biological behavior is equivalent to pathological stage IV, with minimal survival benefits from LN dissection in this area for AEG [2, 8]. However, LN metastases from the cardia region to LPLN has been observed in Siewert type II and III AEG [9]. Furthermore, the extent of LN dissection directly influences the choice between PG and TG for Siewert type II and III AEG, with complications such as anastomotic leakage and reflux esophagitis following surgery being of paramount concern in clinical practice. Additionally, there is increasing emphasis on the relationship between complications and oncological outcomes, as complications can impact adjuvant therapy, thereby affecting oncological prognosis. Therefore, some researchers have advocated for TG with LPLN dissection to ensure oncological safety and lower the risk of reflux esophagitis in patients with Siewert type II and III AEG [10]. In summary, consensus regarding LPLN dissection for Siewert type II and III AEG has yet to be reached.

Thus, this study aims to investigate the risk factors of LPLN metastases and analyze the indications for LPLN dissection for Siewert type II and III AEG.

## Materials and methods

### Patients

**Fig. 1:**
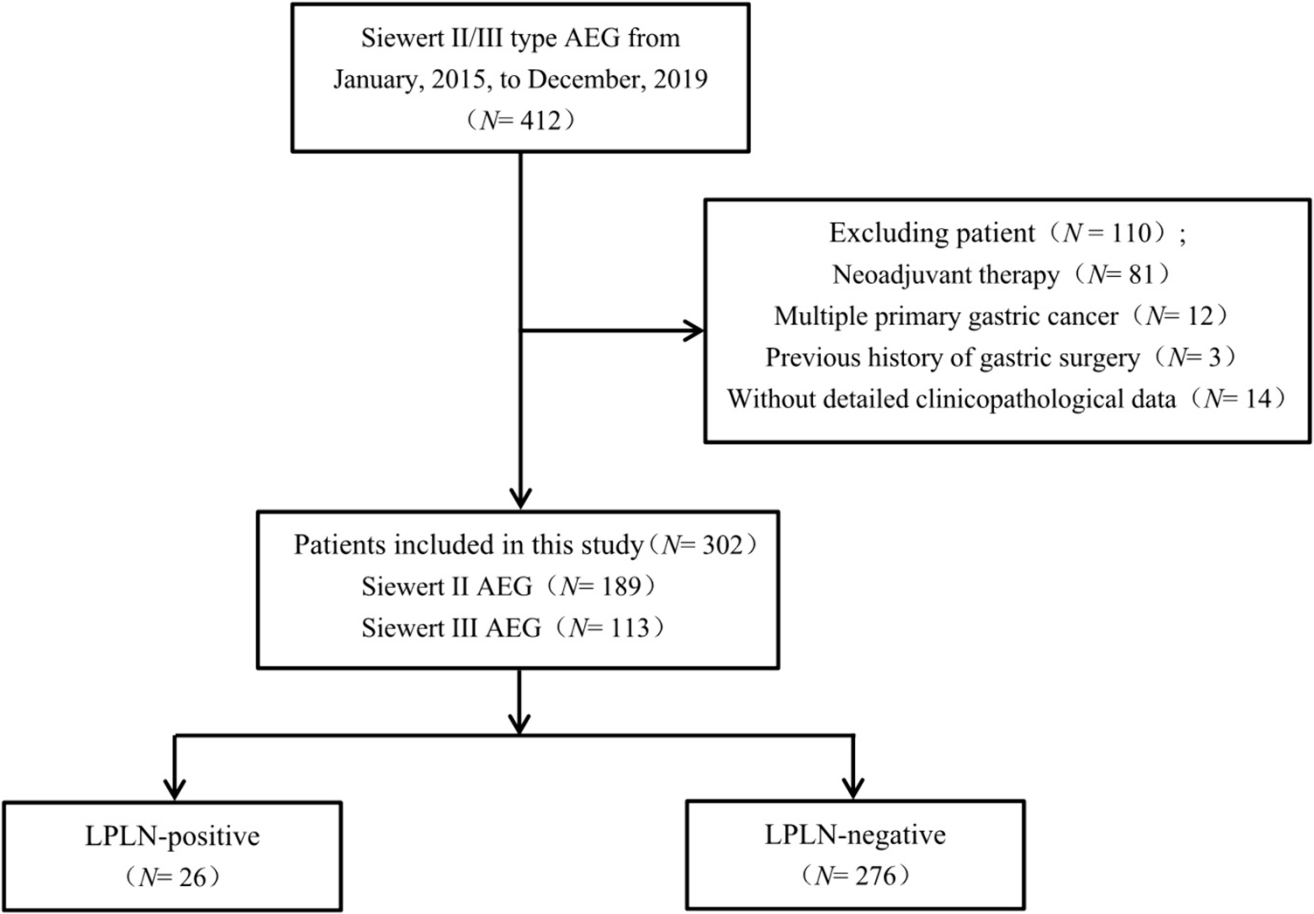
The flowchart of patients with Siewert II and III AEG included in this study

From January 1, 2015, to December 31, 2019, a total of 412 consecutive patients diagnosed with Siewert type II and III AEG underwent either open or laparoscopic TG at the Department of Gastrointestinal Surgery IV, Peking University Cancer Hospital and Institute. Among these patients, 81 individuals who underwent neoadjuvant therapy, 12 with multiple primary gastric cancers, 3 with a history of gastric surgery, and 14 lacking detailed clinicopathological data were excluded from the analysis. Ultimately, the study enrolled 302 patients with primary Siewert type II and III AEG. Prior to surgery, written informed consent was obtained from all patients and their families. This study received approval from the Medical Ethics Committee of Peking University Cancer Hospital (QNJJ2022019). A flowchart detailing the research process is presented in Fig. 1.

### Inclusion and exclusion criteria

The study’s inclusion criteria comprised the following: (1) postoperative pathological confirmation of Siewert type II and III AEG; (2) patients who underwent transabdominal open or laparoscopic TG with D2 LN dissection; (3) postoperative pathology confirming R0 resection; and (4) a minimum of 16 harvested lymph nodes, with each LN station specified in the final pathology report. The exclusion criteria were as follows: (1) patients who received neoadjuvant therapy; (2) patients with multiple primary gastric cancers (≥ 2) or remnant gastric cancer; (3) patients with a history of gastric surgery; and (4) incomplete records of clinicopathologic data.

### Surgical procedure

In our unit, all surgical procedures were performed by three senior gastrointestinal surgeons within the same group, and two expert surgeons routinely conducted thorough searches for lymph nodes following surgery. According to guidelines of the JGCA, all patients underwent radical TG with D2 LN dissection [7]. The extent of lymphadenectomy encompassed stations no. 1, 2, 3, 4sa, 4sb, 4d, 5, 6, 7, 8a, 9, 10, 11p, 11d, and 12a. Dissection of stations no. 19, 20, and 110 lymph nodes were required in cases of esophageal invasion. Histopathological staging was determined according to the 8th edition of the American Joint Committee on Cancer TNM staging criteria [11]. Adjuvant chemotherapy, specifically oxaliplatin combined with S-1, was recommended for patients with the advanced AEG.

### Definitions and research variables

In this study, the diagnosis and classification of Siewert type II and III AEG relied primarily on postoperative specimen anatomy. The LPLN encompassed stations no.4d, 5, 6, and 12a and a positive lymph node in any of these stations was considered as LPLN metastases. To assess the efficacy of LN dissection at specific stations, we employed the concepts of LNM and EI, as proposed by Sasako et al. [12] in 1995. The following formulas were utilized: LNM = (number of patients with LNM / number of patients who underwent LN dissection) × 100 and EI = LNM (%) × 5-year overall survival of patients with metastases (%) / 100. According to the grading system proposed by the JGCA [6, 12], LNM was categorized into three grades: (1) LNM-1 (≥ 10%), indicating strong LN dissection; (2) LNM-2 (5%–10%), suggesting weak LN dissection; and (3) LNM-3 (< 5%), where LN dissection was deemed unnecessary. Similarly, EI was divided into four grades: (1) EI-1 (≥ 5%), indicating strong LN dissection; (2) EI-2 (2%–5%), recommending extensive LN dissection; (3) EI-3 (0.5%–2%), indicating weak LN dissection; and (4) EI-4 (< 0.5%), where LN dissection was unnecessary. In cases where recommendations based on LNM and EI were inconsistent, decisions were made according to the highest standards of EI and LNM in this study.

### Follow-up

All patients were regularly followed up by outpatient visits or telephone interviews with patients or their relative’s following surgery. They were followed up every 3 months within the first 2 years, every 6 months during the subsequent 3 years, and annually thereafter. The follow-up mainly included abdominal and pelvic enhanced computed tomography (CT), chest CT, gastroscopy, and gastrointestinal tumor markers. Overall survival was defined from surgical data until death or loss to follow-up.

### Statistical analyses

Statistical analyses were conducted using SPSS version 26.0 (IBM, Armonk, NY, USA). Quantitative variables were dichotomized according to standard critical values outlined in international guidelines. Patients were categorized into two groups based on their LPLN status: LPLN-positive ( +) and LPLN-negative (–), and compared using either the Chi-square or Fisher’s exact test. Logistic regression models were employed to analyze the risk factors associated with LPLN ( +). Overall survival rate was computed using the Kaplan–Meier method, and group comparisons were made using the log-rank test. Uni- and multivariate survival analyses were performed using Cox regression. Statistical significance was set at *P* < 0.05 for all tests.

## Results

### Clinicopathological variables

A total of 302 patients who underwent TG were included in this retrospective study. Among them, 189 and 113 patients were diagnosed with Siewert type II and III AEG, respectively. The median follow-up duration was 54 months (interquartile range [IQR], 36–108), with the last follow-up conducted on December 31, 2021. When stratified by the status of lymph node metastases in LPLN, 276 patients were categorized as LPLN (-), while 26 patients were classified as LPLN ( +). Carcinoembryonic antigen (CEA), pathological T (pT) stage, lauren type, distance from the esophagogastric junction (EGJ) to the distal end of the tumor, pathological subtype, vascular invasion, nerve invasion, HER-2 expression, and CD34 levels exhibited significant statistical differences (*P* < 0.05) between the two groups (Supplemental Tables 1, 2 and 3). Conversely, variables such as sex, age, body mass index, Siewert type, circumferential distribution, tumor histology, degree of differentiation, presence of concurrent tumors, and immunohistochemical markers did not demonstrate significant differences between the two groups (*P* > 0.05).

**Table 1:** Univariate analysis of risk factors for LPLN metastases in patients with Siewert type II and III AEG

Variables	*P* value	% CI)
Age (years) (≤ 60/ > 60)	0.669	1.194 (0.529–2.698)
Gender(female/male)	0.062	2.343 (0.959–5.724)
Smoking	0.108	2.033 (0.855–4.831)
Alcohol consumption	0.475	1.495 (0.496–4.508)
BMI (kg/m^2^)		
≤ 18.5	0.887	0.240 (0.624–1.491)
18.5–24	0.624	1
≥ 24.0	0.903	1.491 (0.301–7.378)
CEA(−/ +)	0.006	3.119 (1.379–7.058)
CA19-9(−/ +)	0.913	1.073 (0.304–3.795)
C72-4(−/ +)	0.106	2.094 (0.855–5.127)
CA-242(−/ +)	0.371	1.855 (0.480–7.177)
CA12-5(−/ +)	0.115	2.949 (0.769–11.305)
AFP(−/ +)	0.077	3.025 (0.887–10.316)
Siewert type (II/III)	0.338	1.485 (0.661–3.335)
Circumferential distribution (circular/non-circular)	0.691	1.179 (0.523–2.660)
Distance from the EGJ to the distal end of the tumor (cm) (≤ 4.0 / > 4.0)	0.003	4.470 (1.689–11.850)
Lauren type		
Intestinal	0.022	1
Diffuse	0.020	3.820 (1.209–12.070)
Mixed	0.012	3.408 (1.310–8.867)
Pathological type (adenocarcinoma/signet-ring cell)	0.089	2.165 (0.889–5.273)
Tumor differentiation (differentiated/undifferentiated)	0.006	4.62 (1.551–13.760)
Pathological (T) stage (pT1−3/T4)	0.004	4.02 (1.569–10.335)
Vascular invasion (−/ +)	0.017	2.882 (1.212–6.853)
Nerve invasion (−/ +)	0.024	2.968 (1.157–7.617)
Ki-67		
≤ 25%	0.613	1
25–75%	0.353	2.084 (0.443–9.804)
> 75%	0.539	1.623 (0.346–7.613)
HER-2 (−/ +)	0.002	3.717 (1.600–8.631)
EGFR (−/ +)	0.725	1.161 (0.506–2.662)
SALL4 (−/ +)	0.943	1.035 (0.398–2.691)
CD34 (−/ +)	0.034	3.678 (1.106–12.238)
P53 (−/ +)	0.303	0.293 (0.028–3.029)
Cmet (−/ +)	0.818	0.898 (0.359–2.246)
EBER (−/ +)	0.902	1.073 (0.351–3.277)
PD-L1 (−/ +)	0.440	1.375 (0.613–3.085)
MMR (dMMR/pMMR)	0.133	3.565 (0.680–18.683)

*LPLN* lower perigastric lymph node,S *AEG* adenocarcinoma of esophagogastric junction

**Table 2:** Multivariate analysis of risk factors for LPLN metastases in patients with Siewert type II and III AEG

Variables	*P* value	OR (95% CI)		
CEA (−/ +)	0.039	2.806	(1.055–7.462)	
Distance from the EGJ to the distal end of the tumor (cm) (≤ 4.0/ > 4.0)	0.019	4.619	(1.209–9.956)	
Pathological (T) stage (pT1–3/T4)	0.046	2.944	(1.020–8.492)	
Tumor differentiation (differentiated/undifferentiated)	0.294	2.002	(0.547–7.330)	
Lauren type				
Intestinal	0.436		1	
Diffuse	0.891	0.893	(0.176–4.529)	
Mixed	0.304	1.897	(0.560–6.425)	
CD34 (−/ +)	0.282	2.248	(0.513–9.847)	
HER-2 (−/ +)	0.028	3.177	(1.135–8.891)	
Vascular invasion (−/ +)	0.548	1.415	(0.455–4.398)	
Nerve invasion (−/ +)	0.560	1.450	(0.416–5.049)	

*LPLN* lower perigastric lymph node, *AEG* adenocarcinoma of esophagogastric junction

**Table 3:** Univariate and multivariate analysis of the clinicopathologic factors by Cox regression model for patients with Siewert type II and III AEG

Variables	Univariate analysis		Multivariate analysis	
	*P* value	HR(95% CI)	*P* value	HR(95% CI)
Age (years) (≤ 60/ > 60)	0.187	1.334 (0.870–2.048)	–	–
Siewert type (II/III)	0.673	1.096 (0.717–1.674)	–	–
Distance from the EGJ to the distal end of the tumor (cm) (≤ 4.0/ > 4.0)	0.010	1.742 (1.142–2.656)	0.751	1.079 (0.675–1.725)
Tumor differentiation (differentiated/undifferentiated)	0.931	0.982 (0.647–1.490)	–	–
LPLN metastasis (−/ +)	<0.001	2.637(1.534–4.533)	0.044	1.869(1.018–3.431)
Vascular invasion (−/ +)	0.059	1.492 (0.985–2.259)	0.487	1.202 (0.716–2.018)
Nerve invasion (−/ +)	0.408	1.194 (0.785–1.815)	–	–
CEA (−/ +)	0.147	1.392 (0.890–2.178)	–	–
HER-2 (−/ +)	0.068	1.570 (0.967–2.548)	0.316	1.269 (0.761–2.114)
Pathological (T) stage				
≥ T2/T1	0.243	1.863 (0.834–2.642)	–	–
≥ T3/T1–2	0.072	2.447 (1.625–2.532)	0.164	1.834 (0.646–2.334)
T1–3/T4	0.014	1.681 (1.109–2.549)	0.083	0.612 (0.351–1.067)
Pathological (N) stage(pN0–1/N2–3)	0.004	1.828 (1.208–2.766)	0.174	1.315 (0.693–2.199)

*LPLN* lower perigastric lymph node, *AEG* adenocarcinoma of esophagogastric junction

### Univariate analysis of risk factors for LPLN ( +)

Table 1 displays the results of univariate analysis examining risk factors associated with LPLN ( +) in patients diagnosed with Siewert type II and III AEG. Following this analysis, preoperative CEA positivity (OR 3.119, *P* = 0.006), tumor distance from the EGJ (> 4.0 cm) (OR 4.470, *P* = 0.003), tumor differentiation (OR 4.620, *P* = 0.006), pT4 stage (OR 4.020, *P* = 0.004), presence of vascular invasion (OR 2.882, *P* = 0.017), nerve invasion (OR 2.968, *P* = 0.024), HER-2 positivity (OR 3.717, *P* = 0.002), CD34 positivity (OR 3.678, *P* = 0.034), and lauren classification (*P* = 0.022, 0.020, 0.012, respectively) emerged as risk factors for LPLN metastases in Siewert type II and III AEG patients who underwent TG.

### Multivariate analysis of risk factors for LPLN ( +)

Table 2 presents the results of multivariate analysis investigating risk factors associated with LPLN ( +) in patients diagnosed with Siewert type II and III AEG. Independent risk factors for LPLN metastases in Siewert type II and III AEG patients undergoing TG included the distance from the EGJ to the distal end of the tumor (> 4.0 cm) (OR 4.619, *P* = 0.019), CEA positivity (OR 2.806, *P* = 0.039), pT4 stage (OR 2.944, *P* = 0.046), and HER-2 positivity (OR 3.177, *P* = 0.028).

### Analysis of overall survival

**Fig. 2:**
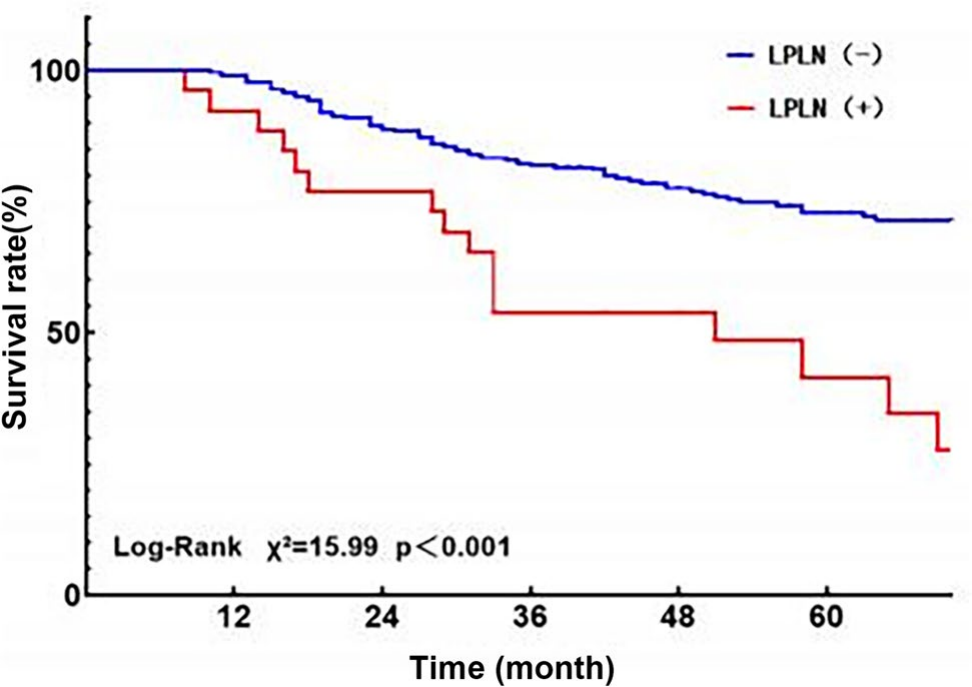
The 5-year overall survival for LPLN-positive and LPLN-negative of Siewert type II and III AEG patients

Table 3 presents the results of uni- and multivariate analyses concerning survival risk factors. In the univariate analysis, factors such as the distance from the esophagogastric junction (EGJ) to the distal end of the tumor (> 4.0 cm) [*P* = 0.010, HR 1.742 (95% CI 1.142–2.656)], pT4 stage [*P* = 0.014, HR 1.681 (95% CI 1.109–2.549)], pathological N (pN) stage [*P* = 0.004, HR 1.828 (95% CI 1.208–2.766)], and LPLN positivity [*P* < 0.001, HR 2.637 (95% CI 1.534–4.533)] emerged as significant risk factors for overall survival in Siewert type II and III AEG patients. To mitigate confounding bias, this study included these variables along with others of clinical significance in the multivariate analysis, revealing that only LPLN metastases remained an independent risk factor for Siewert type II and III AEG patients [*P* = 0.044, HR 1.869 (95% CI 1.018–3.431)]. Notably, the 5-year overall survival rates for Siewert type II and III AEG were 26.9% and 62.3% in the LPLN-positive and LPLN-negative groups, respectively, demonstrating a significant disparity between the cohorts (*P* < 0.001) (Fig. 2).

### LNM and EI of the LN station

Tables 4, 5 and 6 show LNM, 5-year overall survival rate, and EI for LPLN ( +) in patients with Siewert type II and III AEG. Although no. 4d (2.6%, 3.5%, and 3.0%), 5 (2.6%, 3.4%, and 3.3%), 6 (1.6%, 4.4%, and 2.6%), and 12a (1.6%, 2.7%, and 2.0%) LN stations showed lower LNM for patients with Siewert type II, III, and II and III AEG, respectively, the LPLN that comprised these LN stations demonstrated higher LNM, which were 7.4%, 10.6%, and 8.6%, respectively. The 5-year overall survival rate for LPLN ( +) in patients with Siewert III was significantly lower than that in patients with Siewert II AEG (0.00% vs. 21.4%; Table 5). Similar to LNM, although the EI of no. 5 (1.04% and 0.71%), 6 (0.53% and 0.50%), and 12a (0.80% and 0.55%) LN stations were lower for patients with Siewert type II and II and III AEG, respectively, LPLN had higher EI, which were 1.58% and 2.31%, respectively.

**Table 4:** LNM for LPLN metastases in patients with Siewert type II and III AEG

Group	LNM (%)												
	Siewert type			Pathological (T) stage				CEA		Distance from the EGJ to the distal end of the tumor (cm)		HER–2	
	All	II	III	T1	T2	T3	T4	–	+	≤ 4.0	> 4.0	–	+
LPLN	8.6	7.4	10.6	0	0	6.5	13.2	5.9	16.3	6.9	25	6.3	20
No.4d	3.0	2.6	3.5	0	0	1.8	4.9	1.4	7.5	2.9	3.6	2.5	5.5
No.5	3.3	2.6	3.4	0	0	1.8	6.3	2.3	6.3	2.6	10.7	2.1	9.1
No.6	2.6	1.6	4.4	0	0	0.9	4.2	1.8	5.0	1.1	17.9	1.3	9.1
No.12a	2.0	1.6	2.7	0	0	1.8	2.1	1.8	2.5	1.5	7.10	1.6	3.6

*LPLN* lower perigastric lymph node, *AEG* adenocarcinoma of esophagogastric junction, LNM lymph node metastasis

**Table 5:** Five-year OS for LPLN metastases in patients with Siewert type II and III AEG

Group	OS (%)												
	Siewert type			Pathological (T) stage				CEA		Distance from the EGJ to the distal end of the tumor (cm)		HER–2	
	All	II	III	T1	T2	T3	T4	–	+	≤ 4.0	> 4.0	–	+
LPLN	26.9	21.4	0.00	–	–	28.6	15.8	38.5	15.4	31.6	14.3	46.7	18.2
No.4d	0.0	0.00	0.00	–	–	0.0	0.0	33.3	0.00	37.5	0.0	0.0	0.0
No.5	21.4	40	0.00	–	–	50	33.3	60	20	42.9	0.0	60	40.0
No.6	19.4	33.3	0.00	–	–	0.0	16.7	50	25	33.3	20.0	0.0	0.0
No.12a	27.8	50.0	0.00	–	–	50	33.3	25	0.0	50	0.0	50	0.0

*LPLN* lower perigastric lymph node, *AEG* adenocarcinoma of esophagogastric junction, *OS* overall Survival

**Table 6:** EI for LPLN metastases in patients with Siewert type II and III AEG

Group	EI(%)												
	Siewert type			Pathological (T) stage				CEA		Distance from the EGJ to the distal end of the tumor (cm)		HER-2	
	All	II	III	T1	T2	T3	T4	–	+	≤ 4.0	> 4.0	–	+
LPLN	2.31	1.58	0.0	–	–	1.86	2.09	2.27	2.51	1.23	3.55	2.94	3.64
No.4d	0.00	0.00	0.0	–	–	0.00	0.00	0.46	0.00	1.09	0.00	0.00	0.00
No.5	0.71	1.04	0.0	–	–	0.90	2.10	1.38	1.26	1.12	0.00	1.26	3.64
No.6	0.50	0.53	0.0	–	–	0.00	0.70	0.90	1.25	0.37	3.58	0.00	0.00
No.12a	0.55	0.80	0.0	–	–	0.90	0.69	1.00	0.00	0.75	0.00	0.80	0.00

*LPLN* lower perigastric lymph node, *AEG* adenocarcinoma of esophagogastric junction, *EI* efficacy index

### LNM and EI of LN station under subgroup analysis based on independent risk factors for LPLN ( +)

When the subgroup was analyzed by pT stage, the LNM and EI of LPLN were 6.5% and 1.86%, and 13.2% and 2.09% under the pT3 and pT4 stages, respectively. When the subgroup was analyzed by CEA, the LNM and EI of LPLN were 5.9% and 2.27%, and 16.3% and 2.51% under CEA ( −) and CEA ( +), respectively. When the subgroup was analyzed by the distance from the EGJ to the distal end of the tumor, the LNM and EI of LPLN were 6.9% and 1.23%, and 25% and 3.55% under a distance of ≤ 4.0 cm and > 4.0 cm), respectively. When the subgroup was analyzed by HER-2, the LNM and EI of LPLN were 6.3% and 2.94%, and 20% and 3.64% under HER-2 ( −) and HER-2 ( +), respectively.

## Discussion

To date, radical surgery remains the primary treatment for AEG. However, debates persist regarding the extent of gastric resection and the optimal LN dissection for patients with Siewert type II and III AEG still exist [10, 13, 14]. The LPLN, a component of the perigastric LN, significantly influences the choice between PG and TG for Siewert type II and III AEG. This study revealed that several factors, including the distance from the esophagogastric junction (EGJ) to the distal end of the tumor (> 4.0 cm), preoperative carcinoembryonic antigen (CEA) ( +), pT4 stage, and HER-2 ( +), independently correlated with LPLN metastases in patients who underwent TG for Siewert type II and III AEG. Furthermore, LPLN metastasis emerged as an independent predictor of overall survival following TG. Although the overall incidence of LPLN metastasis was below 10%, it exceeded 10% in cases stratified by factors such as the distance from the EGJ to the distal end of the tumor (> 4.0 cm), pT4 stage, CEA ( +), and HER-2 ( +).

CEA, one of the most valuable markers for alimentary system tumors, has been widely used in clinical practice. Several studies have successively reported that higher preoperative CEA levels were associated with perigastric LNM in advanced gastric cancer [15, 16]. Feng et al. [17] and Miki et al. [18] further indicated that CEA could serve for prognostic evaluation, assessing antitumor drugs, and monitoring local recurrence and metastases in gastric cancer. Our study arrived at similar conclusions. The probable molecular mechanisms are as follows: when the gene regulatory program of malignant tumors is impaired, the inhibited CEA gene loses control, resulting in a significant release of CEA into the blood and lymph. An abnormally elevated CEA level could cause cells to lose polarity, leading to disrupted cell junctions and disordered cell arrangement [19]. Simultaneously, it disrupts cell adhesion to collagen and epithelial tissue integrity, facilitating tumor detachment from the primary lesion, thus promoting tumor invasion and metastases. Furthermore, consistent with previous findings [20, 21], our study demonstrated that HER-2 ( +) was positively correlated with LPLN metastases and served as an independent risk factor for LPLN ( +) in Siewert type II and III AEG. Normally inactive in the population, abnormally activated HER-2 can upregulate vascular endothelial growth factor expression, enhancing tumor angiogenesis and cell invasion, ultimately accelerating tumor invasiveness and metastases [22]. Our study reported an HER-2 overexpression rate of 18.2%, significantly higher than the average rate of 12%–13% in China [23]. This difference may be due to the higher proportion of elderly, male, and intestinal-type lauren patients in our study [24]. The LNM of LPLN for Siewert type II and III AEG patients with CEA ( +) and HER-2 ( +) was 16.3% and 20%, with EI of 2.51% and 3.64%, respectively. Following the standards of LNM (> 10%) and EI (> 2%) [12], LPLN dissection should be performed during in surgery.

The involvement of perigastric LN was assessed based on tumor infiltration depth [25], a factor corroborated in our investigation. Our study revealed LNM rates of 0%, 0%, 4.6%, and 29% for LPLN across pathological T stages (T1–4), respectively. The EI of LPLN reached 2.09 for patients with Siewert type II and III AEG with pT4, underscoring the necessity of LPLN dissection during in surgery. This underscores the importance of accurate preoperative clinical T staging, with a remarkable 85.5% consistency observed between preoperative clinical and postoperative pathological T stages, which presented better homogeneity in this study. Therefore, improving the accuracy of clinical T-stage diagnosis is crucial before making surgical decisions. Given its anatomical position, AEG may concurrently invade the proximal esophagus and distal gastric tissue, with the extent of distal gastric tissue invasion significantly correlating with LPLN metastases. Noteworthy studies, including a multicenter retrospective study from Japan, have highlighted the relevance of the distance from the esophagogastric junction (EGJ) to the tumor’s distal end in Siewert type II and III AEG cases. For instance, in patients where the distance from the EGJ to the distal end of the tumor was ≤ 3, 3–5, and > 5 cm, the LNM of LPLN in each group was 2.2%, 8.0%, and 20.0%, respectively [26]. Consequently, PG is advisable when the distance is ≤ 3 cm, while TG is prudent for distances > 5 cm, as suggested by some studies [13, 27]. Notably, LNM rates for LPLN were significantly higher in patients with distances > 4 cm compared to those ≤ 4 cm (25% vs. 6.9%), with corresponding EI values of 3.55 and 1.61, respectively. Therefore, LPLN dissection is warranted for distances > 4 cm, aligning with global research findings [28, 29]. Unlike the JGCA’s recommendation of a 4.0 cm tumor diameter cutoff [7], our study suggests evaluating the EGJ-to-tumor distal end distance to avoid unnecessary LPLN dissection in select Siewert type II and III AEG cases. However, consensus on this distance remains elusive, necessitating further prospective clinical investigations.

Previous studies have indicated associations among undifferentiated tumors [13], signet-ring cells [30], and Siewert type III AEG [10, 13] with metastases to LPLN. However, this study does not definitively confirm such associations. The presence of undifferentiated or signet-ring cells typically signifies a higher malignancy grade, increased invasiveness, and a greater propensity for LN metastases, likely due to lymphatic capillary invasion. Although the rate of LN metastases to the LPLN was higher in patients with Siewert type III AEG compared to type II AEG (10.6% vs. 7.4%), no statistically significant difference was observed in our findings. The inconsistent conclusions may be attributed to factors such as the higher proportion of Siewert type II AEG cases, larger tumor sizes, and more advanced stages of Siewert type III AEG. Consequently, future investigations should prioritize large sample sizes and minimize selection biases for further elucidation of these associations.

In this study, we investigated the incidence of metastasis and the oncological prognosis in patients with Siewert type II and III AEG who underwent TG with LPLN dissection. These findings may offer valuable insights into the selection of gastrectomy and the extent of LN dissection. Consequently, optimizing the surgical approach could potentially mitigate intraoperative and postoperative complications, thereby improving patient prognosis. Nevertheless, our study has several limitations. Firstly, the retrospective nature of the study introduces selection bias that cannot be overlooked. Secondly, the absence of a control group comprising patients who underwent PG without LPLN dissection may compromise the reliability of the conclusions. Thirdly, variations in the degree of concordance between pathological T stage and clinical T stage may diminish the generalizability of the research findings. Finally, the calculation of 5-year overall survival and EI might be inflated in some cases due to incomplete follow-up.

In conclusion, LPLN metastases was an independent risk factor for the prognosis of Siewert type II and III AEG. For patients with preoperative CEA ( +), pT4 stage, HER-2 ( +), and a distance from the EGJ to the distal end of the tumor (> 4.0 cm), TG with LPLN dissection is prioritized for clinical recommendation.

### Supplementary Information

Below is the link to the electronic supplementary material.

Supplementary file1 (DOCX 23 KB)

Supplementary file2 (DOCX 21 KB)

Supplementary file3 (DOCX 18 KB)
