# Supplementary material for: Risk factors and clinical significance of lower perigastric lymph node metastases in Siewert type II and III esophagogastric junction adenocarcinoma: a retrospective cohort study
Source: Surg Endosc. 2024 May 31;38(7):3828–37. doi: 10.1007/s00464-024-10875-y (PMC11219428; doi:10.1007/s00464-024-10875-y)
Supplement: Supplementary file 1 — Supplementary file1 (DOCX 23 KB) [file 464_2024_10875_MOESM1_ESM.docx]

Supplement Table 1. Clinical characteristics of LPLN metastases in patients with Siewert type II and III AEG

| Variables | Total | LPLN（+）  （*N=26*） | LPLN（－）  （*N=276*） | χ² | *P* value |
| --- | --- | --- | --- | --- | --- |
| Gender |  |  |  | 3.665 | 0.097 |
| Female | 54 | 8（30.8） | 44（15.9） |  |  |
| Male | 248 | 18（69.2） | 232（84.1） |  |  |
| Age (years) |  |  |  | 0.183 | 0.669 |
| ≤ 60 | 116 | 11（42.3） | 105（38.0） |  |  |
| >60 | 186 | 15（57.7） | 171（62.0） |  |  |
| BMI (kg/m^2^) |  |  |  | 0.242 | 0.886 |
| ≤18 | 17 | 2（7.7） | 15（5.4） |  |  |
| 18-25 | 134 | 11（42.3） | 123（44.6） |  |  |
| ≥ 25 | 151 | 13（50.0） | 138（50.0） |  |  |
| Smoking |  |  |  | 2.666 | 0.103 |
| No | 163 | 18（69.2） | 145（52.5） |  |  |
| Yes | 139 | 8（30.8） | 131（47.5） |  |  |
| Alcohol consumption |  |  |  | 0.517 | 0.472 |
| No | 239 | 22（84.6） | 217（78.6） |  |  |
| Yes | 63 | 4（15.4） | 59（21.4） |  |  |
| Hypertension |  |  |  | 0.707 | 0.400 |
| No | 223 | 21（80.8） | 202（73.2） |  |  |
| Yes | 79 | 5（19.2） | 74（26.8） |  |  |
| Diabetes |  |  |  | 0.253 | 0.615 |
| No | 270 | 24（92.3） | 246（89.1） |  |  |
| Yes | 32 | 2（7.7） | 30（10.9） |  |  |
| Coronary Heart Disease |  |  |  | 0.170 | 0.559 |
| No | 285 | 25（96.2） | 262（94.2） |  |  |
| Yes | 17 | 1（3.8） | 16（5.8） |  |  |
| CEA (ng/ml) |  |  |  | 8.075 | ***0.004*** |
| ≤ 5.0 | 222 | 13（50.0） | 209（75.7） |  |  |
| > 5.0 | 80 | 13（50.0） | 67（24.3） |  |  |
| CA12-5 (U/ml) |  |  |  | 2.711 | 0.124 |
| ≤ 36 | 248 | 18（85.7） | 230（94.7） |  |  |
| > 35 | 16 | 3 (14.3) | 13（5.3） |  |  |
| CA19-9 (U/ml) |  |  |  | 0.012 | 0.558 |
| ≤ 37 | 266 | 22（88.0） | 244（88.7） |  |  |
| > 37 | 34 | 3（12.0） | 31（11.3） |  |  |
| CA72-4 (U/ml) |  |  |  | 2.710 | 0.088 |
| ≤ 6.7 | 235 | 17（68.0） | 218（81.6） |  |  |
| > 6.7 | 57 | 8（32.0） | 49（18.4） |  |  |
| CA24-2 (U/ml) |  |  |  | 0.824 | 0.408 |
| ≤ 20 | 153 | 12（80.0） | 141（88.1） |  |  |
| > 20 | 22 | 3（20.0） | 19（11.9） |  |  |
| AFP (ng/ml) |  |  |  | 3.406 | 0.084 |
| ≤ 7 | 194 | 11（73.3） | 183（89.3） |  |  |
| > 7 | 26 | 4（26.7） | 22（10.7） |  |  |

Abbreviation: LPLN, Lower perigastric lymph node; BMI, Body mass index, AEG, adenocarcinoma of esophagogastric junction
