# Supplementary material for: Risk factors and clinical significance of lower perigastric lymph node metastases in Siewert type II and III esophagogastric junction adenocarcinoma: a retrospective cohort study
Source: Surg Endosc. 2024 May 31;38(7):3828–37. doi: 10.1007/s00464-024-10875-y (PMC11219428; doi:10.1007/s00464-024-10875-y)
Supplement: Supplementary file 2 — Supplementary file2 (DOCX 21 KB) [file 464_2024_10875_MOESM2_ESM.docx]

Supplement Table 2. Oncological characteristics of LPLN metastases in patients with Siewert type II and III AEG

| Variables | Total | LPLN（+）  （*N=26*） | LPLN（－）  （*N=276*） | χ² | *P* value |
| --- | --- | --- | --- | --- | --- |
| Siewert type |  |  |  | 0.927 | 0.336 |
| Type II | 189 | 14（53.8） | 175（63.4） |  |  |
| Type III | 113 | 12（46.2） | 101（36.6） |  |  |
| Circumferential distribution |  |  |  | 7.054 | 0.133 |
| Circular type | 139 | 11（42.4） | 128（46.4） |  |  |
| Greater curvature | 15 | 1（3.8） | 14（5.1） |  |  |
| Lesser curvature | 91 | 9（34.6） | 82（29.7） |  |  |
| Anterior wall | 17 | 4（15.4） | 13（4.7） |  |  |
| Posterior wall | 40 | 1（3.8） | 39（14.1） |  |  |
| Tumor type |  |  |  | 1.246 | 0.536 |
| Uplift | 25 | 1（3.8） | 24（8.7） |  |  |
| Ulcer | 104 | 11（42.3） | 93（33.7） |  |  |
| Infiltrating | 173 | 14（53.9） | 159（57.6） |  |  |
| Pathological (T) stage |  |  |  | 7.011 | ***0.008*** |
| T1 | 19 | 0（0.0） | 19（6.9） |  |  |
| T2 | 32 | 0（0.0） | 32（10.9） |  |  |
| T3 | 107 | 7（26.9） | 102（37.0） |  |  |
| T4 | 144 | 19（73.1） | 125（45.2） |  |  |
| Lauren type |  |  |  | 8.449 | ***0.015*** |
| Intestinal | 163 | 7（26.9） | 156（56.5） |  |  |
| Diffuse | 41 | 6（23.1） | 35（12.7） |  |  |
| Mixed | 98 | 13（50.0） | 85（30.8） |  |  |
| Tumor differentiation |  |  |  | 3.382 | 0.066 |
| Undifferentiated | 170 | 7（26.9） | 150（54.3） |  |  |
| Differentiated | 132 | 19（73.1） | 126（45.7） |  |  |
| Distance from the EGJ to the distal end of the tumor (cm) |  |  |  | 10.538 | ***0.005*** |
| ≤ 4.0 | 274 | 19（73.1） | 255（92.4） |  |  |
| >4.0 | 28 | 7（26.9） | 21（7.6） |  |  |
| Pathological type |  |  |  | 5.139 | ***0.033*** |
| Signet-ring cell | 55 | 17（65.4） | 46（16.7） |  |  |
| Adenocarcinoma | 247 | 9（34.6） | 230（83.3） |  |  |
| Vascular invasion |  |  |  | 6.166 | ***0.013*** |
| No | 163 | 8（30.8） | 155（56.2） |  |  |
| Yes | 139 | 18（69.2） | 121（43.8） |  |  |
| Nerve invasion |  |  |  | 5.541 | ***0.019*** |
| No | 136 | 6（23.1） | 130（47.1） |  |  |
| Yes | 166 | 20（76.9） | 146（52.9） |  |  |

Abbreviation: LPLN, Lower perigastric lymph node; AEG, adenocarcinoma of esophagogastric junction
