# Supplementary material for: Risk factors and clinical significance of lower perigastric lymph node metastases in Siewert type II and III esophagogastric junction adenocarcinoma: a retrospective cohort study
Source: Surg Endosc. 2024 May 31;38(7):3828–37. doi: 10.1007/s00464-024-10875-y (PMC11219428; doi:10.1007/s00464-024-10875-y)
Supplement: Supplementary file 3 — Supplementary file3 (DOCX 18 KB) [file 464_2024_10875_MOESM3_ESM.docx]

Supplement Table 3. Immunohistochemical characteristics of LPLN metastases in patients with Siewert type II and III AEG

| Variables | Total | LPLN（+）  （*N=26*） | LPLN（－）  （*N=276*） | χ² | *P* value |
| --- | --- | --- | --- | --- | --- |
| HER-2 |  |  |  | 10.366 | ***0.003*** |
| - | 238 | 15（57.7） | 223（83.5） |  |  |
| + | 55 | 11（42.3） | 44（16.5） |  |  |
| EGFR |  |  |  | -0.372 | 0.710 |
| - | 25 | 0（0） | 25（9.6） |  |  |
| + | 48 | 7（26.9） | 41（15.8） |  |  |
| ++ | 112 | 9（34.6） | 103（39.6） |  |  |
| +++ | 101 | 10（38.5） | 91（35.0） |  |  |
| Ki-67 |  |  |  | -0.048 | 0.961 |
| ≤ 25% | 35 | 2（7.6） | 33（13.2） |  |  |
| 25-75% | 107 | 12（46.2） | 95（38.0） |  |  |
| >75% | 134 | 12（46.2） | 122（48.8） |  |  |
| SALL4 |  |  |  | 0.351 | 0.356 |
| - | 234 | 20（76.9） | 214（81.7） |  |  |
| + | 54 | 6（23.1） | 48（18.3） |  |  |
| CD34 |  |  |  | 5.097 | ***0.047*** |
| - | 285 | 22（84.6） | 263（95.3） |  |  |
| + | 17 | 4（15.4） | 13（4.7） |  |  |
| P53 | 51 |  |  | 1.172 | 0.350 |
| - | 25 | 3（75.0） | 22（46.8） |  |  |
| + | 26 | 1（25.0） | 25（53.2） |  |  |
| Cmet |  |  |  | 0.053 | 0.819 |
| - | 73 | 7（28.0） | 66（25.9） |  |  |
| + | 207 | 18（72.0） | 189（74.1） |  |  |
| EBER |  |  |  | 5.188 | 0.078 |
| - | 258 | 24（92.3） | 234（98.7） |  |  |
| + | 5 | 2（7.7） | 3（1.3） |  |  |
| PD-L1 |  |  |  | 0.933 | 0.246 |
| - | 185 | 14（73.7） | 171（82.6） |  |  |
| + | 41 | 5（26.3） | 36（17.4） |  |  |
| MMR |  |  |  | 2.179 | 0.178 |
| dMMR | 8 | 2（7.4） | 6（2.4） |  |  |
| pMMR | 269 | 24（92.6） | 244（97.6） |  |  |

Abbreviation: LPLN, Lower perigastric lymph node; AEG, adenocarcinoma of esophagogastric junction
